# Divinyl Sulfone Cross-Linked Cyclodextrin-Based Polymeric Materials: Synthesis and Applications as Sorbents and Encapsulating Agents

**DOI:** 10.3390/molecules20033565

**Published:** 2015-02-19

**Authors:** Julia Morales-Sanfrutos, Francisco Javier Lopez-Jaramillo, Mahmoud A. A. Elremaily, Fernando Hernández-Mateo, Francisco Santoyo-Gonzalez

**Affiliations:** 1 Departamento de Química Orgánica, Facultad de Ciencias, Instituto de Biotecnología, Universidad de Granada, E1871 Granada, Spain; E-Mails: juliams@ugr.es (J.M.-S.); fhmateo@ugr.es (F.H.-M.); 2 Department of Chemistry, Faculty of Science, Sohag University, Sohag 82524, Egypt; E-Mail: msremaily@yahoo.com

**Keywords:** cyclodextrins, divinyl sulfone, sorbents, encapsulating agents, phenols, curcumin

## Abstract

The aim of this study was to evaluate the crosslinking abilities of divinyl sulfone (DVS) for the preparation of novel water-insoluble cyclodextrin-based polymers (CDPs) capable of forming inclusion complexes with different guest molecules. Reaction of DVS with native α-cyclodextrin (α-CD), β-cyclodextrin (β-CD) and/or starch generates a variety of homo- and hetero-CDPs with different degrees of crosslinking as a function of the reactants’ stoichiometric ratio. The novel materials were characterized by powder X-ray diffraction, electron microscopy and for their sorption of phenol and 4-nitrophenol. They were further evaluated as sorbents with phenolic pollutants (bisphenol A and β-naphthol) and bioactive compounds (the hormone progesterone and curcumin). Data obtained from the inclusion experiments show that the degree of cross-linking has a minor influence on the yield of inclusion complex formation and highlight the important role of the CDs, supporting a sorption process based on the formation of inclusion complexes. In general, the inclusion processes are better described by a Freundlich isotherm although an important number of them can also be fitted to the Langmuir isotherm with R^2^ ≥ 0.9, suggesting a sorption onto a monolayer of homogeneous sites.

## 1. Introduction

Chemical cross-linked cyclodextrin polymers (CDPs) [1] are a novel class of polysaccharide-based materials that have been conceived to utilize the remarkable intrinsic capacity of cyclodextrins (CDs) to form inclusion complexes (ICs) to form advanced networks that synergistically exhibit high sorption properties and particular guest selectivity. Advantages accruing from these capabilities are relevant for the applicability of such materials in diverse fields ranging from remediation technologies [2,3] to drug delivery [4,5], among others.

The removal of organic contaminants from aquatic systems is a crucial issue and recent developments concerning the use of CDPs for environmental purposes have determined their implementation as valuable sorbents [6,7]. In contrast to other materials routinely used in environmental remediation, CDPs are designed to make organic contaminants insoluble and for the enrichment and removal of organic pollutants and heavy metals from soil, atmosphere and water. Overall, the adsorption technique is the best method for the removal of toxic substances from water and is a much better than other physical techniques (flocculation, froth flotation, *etc.*) because of its efficacy and economy [8]. The ability of adsorption to remove toxic chemicals without disturbing the quality of water or leaving behind any toxic degraded products has augmented its usage in comparison to electrochemical, biochemical or photochemical degradation processes. A wide variety of solids ranging from synthetic materials to others of natural origin such as polysaccharides have been employed as sorbents in this technique. CDs have recently been the object of numerous modifications and polymerization studies in the search for non-conventional but cheaper and more effective sorbents. The easy enzymatic preparation of CDs from starch and the presence of a central hydrophobic cavity in their structures, making them ideal hosts in the formation of ICs of aromatics and other hydrophobic organic pollutants, are advantageous characteristics. Nonetheless, because of their water solubility native CDs must be unavoidably processed into water-insoluble solid forms (CDPs) in order to be implemented as usable sorption materials.

On the other hand, CDPs are opening new perspectives in pharmacotherapy where they are emerging as advanced delivery systems [4,5,9,10,11]. In a broad sense, native and chemically derivatized CDs have emerged as an important tool in the formulator’s armamentarium as ideal assistants to overcome the most relevant challenges of the formulation of both old drugs and novel biopharmaceuticals, namely the balance between solubility, stability and permeability [9,12]. Encapsulation of a guest drug inside the CD by the formation of ICs provides an efficient hydrophilic camouflage that produces remarkable physicochemical consequences. For example, an improvement in the apparent solubility and dissolution rate in the case of poorly water-soluble drug candidates [13]. Interestingly, CDs can cooperatively work when they are close together, provoking a displacement in the complex formation equilibrium towards the ICs [14]. For this reason, CDs have been used as outstanding building blocks for the development of novel supramolecular drug delivery systems such as poly(pseudo)rotaxanes [11], polymer-CD grafted and polymer-guest grafted physical networks [15] and also chemically cross-linked CDPs [1].

The most direct and general method for the synthesis of cross-linked CDPs is by reacting the hydroxyl groups of native CDs with bi- or multi-functional molecules (cross-linker agents) to form stable cross-linked networks. Epoxides, isocyanates, active carbonyl compounds, polycarboxylic acids and some of their activated derivatives (chlorides and anhydrides) as well as aldehydes are among the cross-linkers reported for the preparation of CDPs. The most thoroughly studied CD polymerization is the cross-linking reaction with the epoxide epichlorohydrin (EPI) [16] although ethylene glycol diglycidyl ether (EGDE) has emerged as a non-toxic and environmentally friendly alternative to the diepoxide counterpart. Diisocyanates—toluene diisocyanate (TDI) and hexamethylene diisocyanate (HDI)—are similarly useful reagents to obtain CDPs, but they usually require organic solvents as reaction media [17,18] On the other hand, active carbonyl compounds, such as N,N-carbonyldiimidazole (CDI) and diphenylcarbonate (DPC), have been utilised as cross-linkers to generate CDPs. Finally, polycarboxylic acids and some of their halides and anhydride derivatives have been reported as “eco-friendly” cross-linkers (particularly in the case of citric acid) being applied with success in the preparation of water-insoluble CDPs [19,20,21].

It should be mentioned that the already well-established capabilities of divinyl sulfone (DVS) to act as a non-zero length cross-linker have never been exploited for the preparation of CDPs. Vinyl sulfones readily undergo conjugate additions as excellent Michael acceptors because of the electron poor nature of their double bond [22,23]. A significant body of work has been devoted to the conjugate additions of vinyl sulfones with carbon nucleophiles, but other heteroatomic nucleophiles involving nitrogen, sulfur and oxygen can also participate efficiently in these reactions in a protic environment where the use of a base catalysis is necessary, except in the case of amines. Among the different bisvinyl sulfones, DVS is the most popular cross-linker reagent because of its reactivity, stability, solubility in water and affordable price, having found a plethora of applications in bioconjugation [24], proteomics [25] and also in the preparation of polysaccharide-based networks. In this latter case, the properties of the electrophilic double bond of DVS have attracted research into the crosslinking of diverse natural polysaccharides (cellulose, dextran, agarose and hyaluronic acid). Efforts have been directed mainly for the preparation of hydrogel-based materials with improved mechanical and chemical properties to be used as advanced materials in a variety of applications: chromatographic supports, controlled drug-delivery systems and biocompatible biomaterials for biomedical applications.

In the present work the potential of DVS as a cross-linker is exploited for the preparation of novel water-insoluble homo- and hetero-CDPs by cross-linking native α-CD, β-CD and starch [26]. The capabilities of the novel materials as sorbents for the removal of a variety of some representative phenolic pollutants and as encapsulating agents for bioactive compounds such as a model steroid hormone (progesterone) and a naturally phenolic compound (curcumin) are also explored and reported.

## 2. Results and Discussion

### 2.1. Preparation and Characterization of DVS Cross-Linked Polymers 

**Scheme 1:**
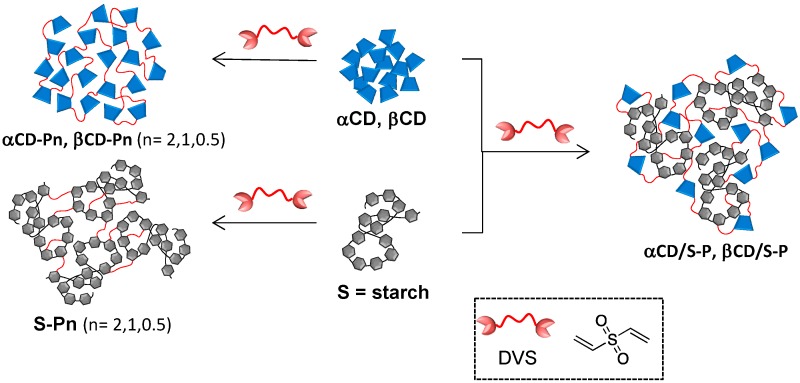
Synthesis of DVS cross-linked homo- (**αCD-Pn**, **βCD-Pn**, **S-Pn**) and hetero-polymers (**αCD/S-P** and **βCDS-P**); *n* = stoichiometry DVS:Glc ratio: *n* = 2 (2:1), *n* = 1 (1:1), *n* = 0.5 (0.5:1).

With the aim of preparing novel CD-based sorbents, α- and β-CD were chosen as starting materials for accessing CDPs polymers synthesized using DVS as cross-linking reagent as shown in Scheme 1 [26]. It is well established that the electron-deficient double bond of the vinyl sulfone groups in DVS is reactive toward the OH groups present in natural linear polysaccharides (cellulose, dextran, agarose and hyaluronic acid), a reaction that usually occurs under alkaline conditions. Considering that the pK_a_ of the OH groups of the Glc units in CD is about 12–13, carbonate buffer (pH 12.0) was chosen to perform their deprotonation to yield alkoxide ions. The primary OH groups at the C-6 position exhibit a higher reactivity with respect to the secondary OH groups. Being active nucleophiles, the alkoxide groups form covalent linkages with the electrophilic double bonds of DVS. Different DVS:Glc stoichiometries and reaction times were tested to ensure the reaction of all the vinyl sulfone groups. ^1^H-NMR analysis revealed that, regardless of the DVS:Glc stoichiometry, the vinyl signal vanished after 7–8 h of reaction at room temperature.

CDs are well known for their capability to form inclusion complexes and their cross-linking yields polymers that exhibit high sorption properties and particular guest selectivity. In order to have a reference material without such capability, starch was cross-linked with DVS under similar conditions. However, owing to the poor solubility of starch in the carbonate buffer, the solution required heating until the starch was completely dissolved, followed by subsequent addition of DVS once the system reached room temperature. Additionally to these reference materials and the CDs-based homopolymers, heteropolymers with expected intermediate features were also synthesized by cross-linking α or β-CD and starch (1:1 w/w) with DVS (1:1 DVS: Glc stoichiometry).

As depicted in Table 1, there is a clear relation between the DVS:Glc stoichiometry and sulphur content (*i.e.*, the degree of cross-linking). Thus, regardless of the polysaccharide, both homo- and hetero-polymers with stoichiometry 1:1 yielded the same amount of material with a similar degree of cross-linking (23%–27% S/C). As expected, the content of sulphur for S-P0.5 (19%) and **αCD-P2** (33%) is lower and higher respectively.

**Table 1:** Features of DVS cross-linked homo- (**S-Pn** and **CD-Pn**) and hetero-polymers (**CDS/S-P**).

Polymer	Stoichiometry DVS:Glc	Elemental Analysis (% S/C)	Mass Resulting from the Reaction (gr)
**S-P1 ^a^**	1:1	26.2	4.1
**S-P0.5 ^a^**	0.5:1	19.4	3.7
**βCD-P1 ^a^**	1:1	26.7	3.8
**αCDP2 ^a^**	2:1	33.3	5.4
**αCDP1 ^a^**	1:1	27.2	3.8
**βCD/S-P**	1:1 ^b^	22.6	3.9
**αCD/S-P**	1:1 ^b^	27.2	3.8

^a^ S = starch; *n* = stoichiometry DVS:Glc ratio: *n* = 0.5 (0.5:1); *n* = 1 (1:1), *n* = 2 (2:1); ^b^ Amount of Glc resulting from a 1:1 (w/w) combination of CD and starch.

**Figure 1:**
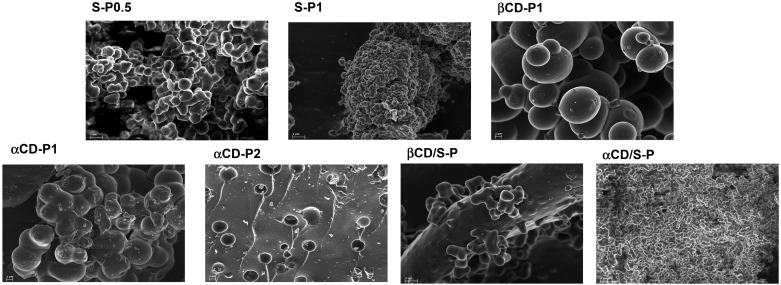
Electron microscopy study: Micrographs taken at 20,000× (**S-P0.5**, **S-P1** and **αCD/S-P**) and 10,000× (**βCD-P1**, **αCD-P1**, **αCD-P2** and **βCD/S-P**) magnifications.

The prepared materials were characterized by ATR-IR, powder X-ray, SEM, TGA and elemental analysis. ATR-IR spectrum are almost identical for all compounds without remarkable differences. (Supplementary Material, Figure S1) Powder X-ray diffraction patterns show a broad hump at 2θ = 13–23° that indicates that the polymers existed in a form of non-crystalline state (Supplementary Material, Figure S2). The SEM images (Figure 1: 20,000× and 10,000× magnifications) reveal the nature of the surface of the dry polymers, showing that 1:1 stoichiometry homopolymers made of α- or β-CD are lobular materials that share similar features, where the degree of cross-linking exerts a clear influence. This is especially relevant for the case of **αCD-P2** whose surface is full of holes. The surface of the heteropolymer **αCD/S-P** resembles that for S-P0.5 whereas **βCD/S-P** shows a more homogeneous surface.

TGA under a nitrogen atmosphere (Supplementary Material, Figure S3) shows that polymers absorb ambient moisture and thermal degradation starts around 330 °C to reach the maximum rate between 335.3 °C for S-P0.5 to 376.6 °C for **βCD-P1**. At 950 °C the decomposition is not total, remaining a residual mass that ranges from 8.8% to 12.2% (Table 2).

**Table 2:** Main features of DVS cross-linked homo- (**S-Pn** and **CD-Pn**) and hetero-polymers (**CDS/S-P**) calculated from the TGA analysis in a nitrogen atmosphere.

Polymer	Water Content	Onset T (To)	DTG (Tp)	Residual mass
**S-P1**	1.43%	335 °C	365.36 °C	10.55%
**S-P0.5**	1.96%	325 °C	335.31 °C	11.79%
**βCD-P1**	2.85%	340 °C	376.65 °C	10.99%
**αCD-P2**	1.75%	330 °C	352.20 °C	10.38%
**αCD-P1**	2.72%	330 °C	373.94 °C	12.25%
**βCD/S-P**	2.25%	335 °C	354.06 °C	8.77%
**αCD/S-P**	1.35%	330 °C	361.11 °C	9.73%

**Figure 2:**
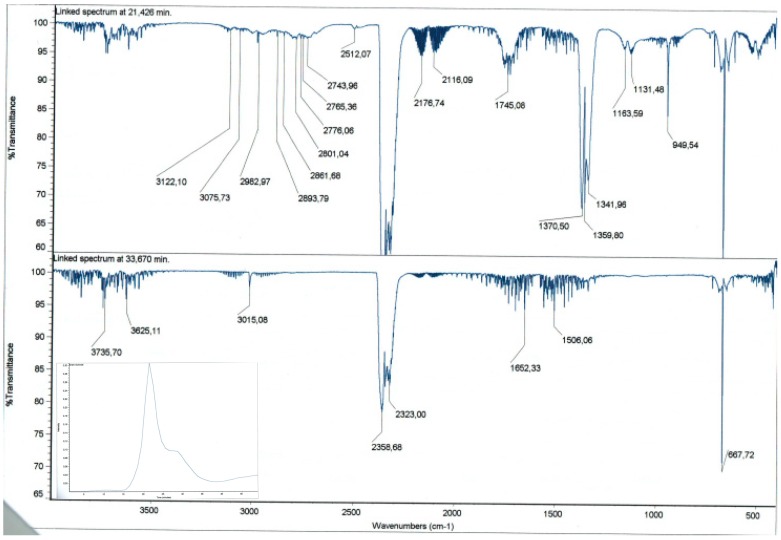
Representative TGA-IR result. Spectra at two single time points 21.4 min and 33.7 min that are shown in the Gram-Schmidt profile (insert).

The analysis of the chemical species formed during the thermal decomposition reveals the presence of CO_2_ (signals at 3736 cm^−1^, 3625 cm^−1^, 2359 cm^−1^, 2323 cm^−1^, 668 cm^−1^), CO (signals at 2177 cm^−1^, 2116 cm^−1^), SO_2_ (signals at 1370 cm^−1^, 1342 cm^−1^, 1163 cm^−1^), CH_4_ (signal at 3015 cm^−1^), methanal (signals at 2894 cm^−1^, 2861 cm^−1^, 2801 cm^−1^, 2744 cm^−1^, 1745 cm^−1^) and probably ethene (signals at 3122 cm^−1^, 3076 cm^−1^, 2983 cm^−1^, 1360 cm^−1^) (Figure 2).

**Figure 3:**
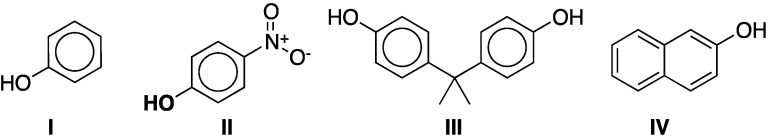
Model phenolic compounds phenol (**I**) and 4-nitrophenol (**II**) and representative phenolic pollutants bisphenol-A (**III**) and β-naphthol (**IV**).

As an additional characterization, the sorption of phenol (**I**) and 4-nitrophenol (**II**) (Figure 3) was studied. They were chosen as model compounds based on the importance of phenolic compounds as toxic pollutants commonly encountered in trace quantities in aqueous effluents from various manufacturing processes such as oil refineries, coke plants, and phenolic resin plants. In addition, the binding of phenol and 4-nitrophenol to cyclodextrin has been studied by other authors [27,28,29,30,31,32] and the thermodynamics of the inclusion complexes are well known [33].

Prior to the study of the sorbent capacities of the synthesized CDPs towards these compounds, the kinetics of the sorption process were investigated to ascertain the time required to reach the equilibrium in that dynamic process. In these assays, the sorption capacity of the polymers was evaluated by measuring the remaining amounts of those compounds in solution *versus* the contact time when a fixed concentration (100 ppm) of the organic pollutants was stirred in the presence of an identical amount of the different polymers. The data showed that a plateau is reached after only 15–30 min independently of the polymer structure, time that is indicative of fast sorption kinetics in all cases.

The sorption capacities were next studied by adding identical volumes of solutions of the model phenolic pollutants with concentrations ranging from 20–5000 ppm to a fixed amount (100 mg) of these materials (Table 3). The quantity of the pollutant adsorbed was determined by measuring the UV-Vis absorption in the solution. Data were fitted to the linearized forms of the Langmuir isotherm (C_e_/Cq = 1/K_L_ + 1/q_max_ × Ce) and the Freundlich isotherm (LnCq = LnK_F_ + (1/N) × LnCe) (Supplementary Material, Figures S4 and S5). It is important to recall that the Langmuir model assumes a monolayer sorption of solute on homogeneous sorption sites, being Ce and Cq the concentration of solute at the equilibrium in the solution and in the polymer, respectively, K_L_ the bonding energy constant and q_max_ the sorption maximum. By contrast, the Freundlich model relies on an empirical expression that can be applied to non-ideal sorption on dissimilar surfaces along with multilayer absorption, being Ce concentration of solute at the equilibrium in the solution, K_F_ the capacity factor and N is a parameter related to the heterogeneity of the system [34].

The determination coefficients reveal that the process of sorption is better described by the isotherm of Freundlich (Table 3), particularly for the polymers obtained by cross-linking of starch that showed low affinity for both phenol and 4-nitrophenol when compared to the other polymers. In general, the degree of cross-linking does not seem to play a significant influence on the properties of the material. As expected, Langmuir and Freundlich fittings demonstrate, first, that the homo-CDPs are the best materials. In addition, the results with the hetero-CDPs are outstanding when compared to those obtained from starch, highlighting the role of CD in the sorption, and suggesting the formation of inclusion complexes that may explain the poor performance of the cross-linked starch. According to the thermodynamic parameters reported in literature, α-CD and β-CD bind more strongly to phenol and 4-nitrophenol, respectively [33] and this is in full agreement with the K_L_ values estimated for the heteropolymers. However, it is also apparent that α-CD homopolymer has the highest affinity and capacity factor for 4-nitrophenol and this discrepancy may be rationalized considering that the sorption process on CD in solution is different to that when it forms an insoluble polymer.

### 2.2. Sorption of Representative Phenolic Pollutants β-Naphthol *(**III**)* and Bisphenol-A *(**IV**)*

As discussed above phenolic compounds were chosen as model systems because they are important toxic pollutants. Due to increasingly stringent restrictions for these pollutants, a panel of diverse techniques has been proposed for their removal: liquid-liquid extraction, activated carbon, anion-exchange resins, micellar-enhanced ultrafiltration, use of membranes by pervaporation, and also the use of CDPs. In the reported cases related with the use of CDPs for the removal of phenolic compounds, CDs has been cross-linked by using EPI and diisocyanates as cross-linker reagents having generally showed good removal efficiency.

In order to test the performance of the polymers they were assayed with two representative phenolic pollutants with larger molecular dimensions than the models compounds: bisphenol A (**III**) and β-naphthol (**IV**) (Figure 3). Bisphenol A (BPA) is used in the manufacture of epoxy resins and polycarbonate. BPA has been identified as an endocrine-disrupting chemical by the US Environmental Protection Agency, and the World Wide Fund for Nature [35,36] that affects the reproductive behaviour of both humans and animals and induces various diseases including cancer [37]. The poor solubility of this compound makes BPA difficult to remove and it bioaccumulates [38]. Naphthalene derivatives with substituents at position 2 are usually more toxic than those at position 1, therefore, β-naphthol is listed among top priority contaminants [39]. This compound biodegrades very slowly and, due to its toxicity, industrial wastewater containing 2-naphthol must be treated before it is reused or discharged to the environment.

As these compounds are poorly water-soluble, MeOH and DMSO were used as co-solvents (10% v/v) and the solutions were prepared in a more restricted concentration range (20–1000 ppm). Considering that the results from the sorption of phenol and 4-nitrophenol suggest that the degree of cross-linking plays a minor role (Table 3), only polymers with 1:1 DVS:Glc stoichiometry were tested. The sorption capacities were evaluated following the analytical methodology described above and the main parameters of the isotherms are depicted in Table 4. In the present case, the BPA sorption process is, in general, better described by a Freundlich isotherm, being the polymer based on cross-linked starch the material that shows the poorest performance. However, the determination coefficients reveal that data of the sorption from solutions containing 10% (v/v) MeOH on **βCD-P1**, **αCD-P1** and **βCD/S-P** also fit very well the Langmuir isotherm, suggesting that the process can be described as a sorption onto a monolayer of homogeneous sites. The estimated maximum sorption is in the range of the 84 mg/g described for cross-linked CD with EPI [40], except for **αCD-P1** which is unexpectedly high because according to bibliography the improvement in the solubility of BPA in water by addition of hydroxypropyl-α–CD is worse than that by hydroxypropyl-β-CD [41]. This result may attributed to the polymeric (*i.e.*, multivalent) nature of **αCD-P1**. Interestingly, ^1^H-NMR studies on the inclusion complexes of BPA with α–CD did not find any signal that could be assigned to the BPA protons [42] and this may be rationalized as the formation of a week inclusion complex that is in full agreement with the low K_L_ values estimated for the polymers containing α-CD. The isotherm of Langmuir can be used to describe the sorption of BPA onto **βCD-P1** and allows the analysis of the co-solvent on the process: the estimated maximum capacity remains constant whereas the bonding energy constant diminishes when the co-solvent is polar and aprotic.

**Table 3:** Freundlich and Langmuir parameters for the sorption of phenol (**I**) and 4-nitrophenol (**II**) (20–5000 ppm solutions in water) on the DVS cross-linked homo- (**S-Pn** and **CD-Pn**) ^a^ and heteropolymers (**CDS/S-Pn**) ^a^ (100 mg). (Supplementary Material, Figures S4 and S5).

Isotherm	Parameter	S-P1 ^a^		S-P0.5 ^a^		βCD-P1 ^a^		αCDP2 ^a^		αCDP1 ^a^		βCD/S-P ^a^		αCD/S-P ^a^	
		I	II	I	II	I	II	I	II	I	II	I	II	I	II
Freundlich	R^2^	0.993	0.999	0.983	0.987	0.993	1.000	0.992	0.998	0.986	0.994	0,997	0.999	0.997	0.999
	N	0.887	0.999	0.857	1.077	1.507	1.326	1.430	1.515	1.863	1.575	1.1248	1.496	1.445	1.319
	K_F_ × 10^3^	2.151	19.111	1.206	23.004	199.149	340.003	243.777	627.696	623.005	902.217	56.067	404.501	146.413	193.438
Langmuir	R^2^	0.088	0.063	0.383	0.010	0.844	0.816	0.895	0.867	0.858	0.921	0.771	0.817	0.807	0.749
	K_L_	n/a	n/a	n/a	n/a	0.040	0.119	0.064	0,150	0.066	0.225	0.021	0.083	0.032	0.060
	q_max_ (mg/g)	n/a	n/a	n/a	n/a	67.788	200.275	97.362	148.723	61.667	148.643	78.724	135.896	66.112	164.027

^a^ S = starch; *n* = stoichiometry DVS:Glc ratio: *n* = 0.5 (0.5:1); *n* = 1 (1:1); *n* = 2 (2:1).

**Table 4:** Freundlich and Langmuir parameters for the sorption of bisphenol A (**III**) and β-naphthol (**IV**) (20–1000 ppm solutions in water and either 10% methanol or 10% DMSO) on the DVS cross-linked homo- (**S-Pn** and **CD-Pn**) ^a^ and hetero-polymers (**CDS/S-P1**) (1:1 DVS:Glc stoichiometry) (100 mg). (Supplementary Material, Figures S6–S9).

Solvent	Isotherm	Parameter	S-P1 ^a^		βCD-P1 ^a^		αCDP1 ^a^		βCD/S-P ^a^		αCD/S-P ^a^	
			III	IV	III	IV	III	IV	III	IV	III	IV
MeOH	Freundlich	R^2^	0.998	0.998	0.970	0.998	0.995	0.998	0.959	0.996	0.993	0.993
		N	0.620	1.016	1.554	1.457	1.164	1.451	1.542	1.383	1.065	1.240
		K_F_ × 10^3^	57.487	66.497	1417.224	725.786	339.596	752.014	1631.827	390.589	199.708	240.918
	Langmuir	R^2^	0.578	0.308	0.972	0.883	0.980	0.838	0.966	0.840	0.790	0.874
		K_L_	n/a	n/a	0.707	0.295	0.238	0.313	0.856	0.169	0.173	0.136
		q_max_ (mg/g)	n/a	n/a	69.153	72.826	119.317	75.043	76.231	56.642	190.905	67.717
DMSO	Freundlich	R^2^	0.997	0.997	0.984	0.996	0.995	0.992	0.944	1.000	0.999	0.994
		N	0.927	1.068	1.459	1.298	1.066	1.291	1.290	1.234	1.050	1.159
		K_F_ × 10^3^	32.998	43.205	865.455	309.468	159.151	360.703	598.517	161.121	105.484	117.209
	Langmuir	R^2^	0.271	0.872	0.947	0.902	0.838	0.944	0.850	0.822	0.747	0.944
		K_L_	n/a	0.035	0.403	0.135	0.135	0.192	0.341	0.838	0.091	0.076
		q_max_ (mg/g)	n/a	80.137	69.844	66.944	185.832	67.727	88.838	0.838	256.673	66.944

^a^ S = starch; *n* =1 (1:1) stoichiometry DVS:Glc ratio.

Data for the sorption of β-naphthol follow the expected trend. They can be fitted to the Freundlich isotherm and the polymer obtained by crosslinking of starch shows the worst performance, highlighting the importance of the formation of inclusion complexes (Table 4). The Langmuir isotherm can describe the sorption of β-naphthol in 10% DMSO on DVS cross-linked α,β-CD-polymers, suggesting that there is not a clear influence of the size of the CD on the maximum estimated capacity and on the bonding energy constant.

### 2.3. Sorption of Bioactive Compounds

**Figure 4:**
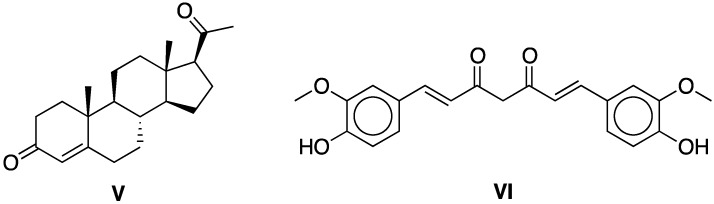
Archetypal drug for encapsulation assays: Progesterone (**V**) and curcumin (**VI**)

As mentioned above, CDPs are attractive materials in the medicinal and pharmaceutical field as encapsulating agents with a high potential in the formulation of drugs and biopharmaceuticals. To evaluate these inclusion capabilities in the case of the DVS cross-linked CDPs a representative steroid hormone, progesterone (**V**), and a naturally bioactive phenolic compound, curcumin (**VI**), were chosen as archetypal models (Figure 4).

Progesterone is a lipophilic drug used in menopausal hormone replacement therapy and reproductive control functions in which oral delivery is limited due to its poor aqueous solubility. In addition, progesterone exhibits a low tolerance when administered in high doses. Efforts to overcome these undesirable properties have led to the development of suitable carriers to facilitate the drug administration to the target site while optimizing delivery amount over a predetermined period [43]. Among the CD-based systems used for delivery progesterone different approaches have been undertaken such as the formation of nanoparticles from amphiphilic CDs [44,45,46], incorporation of progesterone-CD ICs into ternary systems [solid lipid nanospheres [47], bovine serum albumin nanospheres [48], polymeric nanoparticles [49] and hydrophilic polymers–chitosan [50], PEG [51,52] and others [53] and molecular imprinting of CDPs [54].

Curcumin is a natural yellow phenolic antioxidant of low toxicity that shows pharmacological activity on various diseases such as cancer, cardiovascular disease, inflammatory bowel syndrome, wound healing, Alzheimer’s disease, rheumatoid arthritis, and diabetes. Despite these advantages, applications of curcumin are limited because of its low bioavailability, rapid degradation and metabolism. Many studies directed to overcome these problems have been focused on the development of nanopreparations enabling encapsulation solutions [55], and, nowadays, liposomes, polymeric nanoparticles and micelles, conjugates, peptide carriers, solid dispersions, lipid nanoparticles, emulsions, and also CDs have been explored for these purposes [56,57,58]. The application of CDs as nano-carrier systems has been demonstrated to be a viable choice either by the use of native or chemical modified CDs as well as by the application of a β-CDPs in one reported case [59].

#### 2.3.1. Sorption of Progesterone (**V**)

To study the encapsulation of progesterone by the DVS cross-linked CDPs, solutions of those compounds using MeOH (10%) as co-solvent (10%) were prepared considering their low water solubility and the results obtained for the phenolic pollutants. In these conditions, the highest solubility attained was 50 ppm and, on this basis, the solutions ranged from 5 to 50 ppm (Table 5). The encapsulation capacities of the polymers with 1:1 DVS:Glc stoichiometry were assayed by the batch methodology described above. As shown in Table 5 experimental data fit the Freundlich isotherm and the estimated values of K_F_ point to **βCD/S-P** as the most promising encapsulating agent and suggest the suitability of the carbohydrate in the rank order β-CD > starch > α-CD. The Langmuir isotherm can be applied to **βCD-P1** and **βCD/S-P** and allows the evaluation of the contribution of the starch. Interestingly the better values of both K_L_ and q_max_ for **βCD/S-P** confirm the positive contribution of the starch to the encapsulating properties of the heteropolymers.

**Table 5:** Freundlich and Langmuir parameters for the sorption of progesterone (**V**) (5–50 ppm in methanol:water 1:9) and curcumin (**VI**) (2–16.7 mM in ethanol) on the DVS cross-linked homo- (**S-P1** and **CD-P1**) and heteropolymers (**CDS/S-P1**) (1:1 DVS:Glc stoichiometry) (100 mg and 125 mg respectively). (Supplementary Material, Figures S10 and S11).

Isotherm	Parameter	S-P1		βCD-P1		αCD-P1			βCD/S-P		αCD/S-P	
		V	V		VI		V	V		VI		V
Freundlich	R^2^	0.992	0.993		0.974		0.992	0.994		0.999		0.981
	N	1.171	1.222		1.036		1.118	1.214		0.985		1.198
	K_F_ × 10^3^	114.040	291.825		10,411.744		84.983	442.241		8.915		110.339
Langmuir	R^2^	0.751	0.972		0.275		0.755	0.974		0.107		0.647
	K_L_	0.099	0.281		na		0.077	0.452		na		0.093
	q_max_ (mg/g)	5.807	6.645		na		6.480	7.928		na		5.074

#### 2.3.2. Sorption of Curcumin

The study of the encapsulation of curcumin was limited to the materials containing β-CD and 1:1 Glc:DVS stoichiometry. As depicted in Table 5 data could only be fitted to the Freundlich isotherm, suggesting that the process involves a non-ideal sorption on dissimilar surfaces and/or multilayer sorption. The value of K_F_ estimated for **βCD-P1** is several orders of magnitude higher than that for **βCD/S-P**. This difference may be rationalized on the basis of the proposed structure of the 2:1 CD:curcumin inclusion complex [60] where the distance between CD plays a critical role in the stability of the complex, in **βCD-P1** being at more favourable positions than in **βCD/S-P** to yield the formation of the 2:1 ICs.

## 3. Experimental Section

### 3.1. Materials

Native cyclodextrins α and β-cyclodextrin (α- and β-CD) with 95% degree of purity were bought from TCI Europa N.V. (Madrid, Spain). Divinyl sulfone (DVS) with 97% degree of purity was purchased from AlfaAesar (Karlsruhe, Germany) Phenolic compounds (phenol, 4-nitrophenol, bisphenol, 2-naphthol), progesterone and curcumin were acquired from Sigma-Aldrich (Madrid, Spain). Ethanol, methanol and dimethyl sulfoxide were of analytical grade reagents (Panreac Quimica, Barcelona, Spain) and were used without further purification.

### 3.2. Synthesis of DVS Cross-Linked Homo-Polymers (S-Pn and CD-Pn) 

A solution of the corresponding oligo- (α- or β-CD) or polysaccharide (starch) was prepared by dissolving these materials in carbonate buffer (0.5 M, pH 12, 150 mL) under magnetic stirring at room temperature, for the case of α- or β-CD, or by heating under reflux (15–20 min) for the case of starch. After magnetic stirring for additional 30 min, DVS was added to yield 0.5:1, 1:1 or 2:1 DVS:glucose (DVS:Glc) (*n* = 0.5, 1 or 2, respectively) stoichiometric molar ratios. The reaction was maintained for 7–8 h giving as a result a white solid powder that precipitates from the reaction media. In order to remove unreacted components and neutralize the pH, the resulting polymer was filtered and thoroughly washed with deionized water (4 L) and then with MeOH (0.5 L) and diethyl ether (0.25 L). The resulting cross-linked polymer (**S-Pn** or **CD-Pn**) was dried *in vacuo* for 18 h at 50 °C [26].

### 3.3. Synthesis of DVs Cross-Linked Hetero-CDPs (CDS/S-P)

A suspension of starch (1.5 g) in carbonate buffer (0.5 M, pH 12, 150 mL) was heated under reflux until complete solubilization (15–20 min). The solution was left to reach room temperature and α- or β-CD (1.5 g) was added and dissolved under magnetic stirring. The stirring was continued for 30 min prior to the addition of DVS (2 mL) and maintained during the cross-linking reaction (7–8 h) giving as a result a white solid powder that precipitates from the reaction media. In order to remove unreacted components and neutralize the pH, the resulting polymer was filtered and thoroughly washed with deionized water, MeOH and diethyl ether. The resulting cross-linked starch-CD polymer **CDS/S-P** was dried *in vacuo* for 18 h at 50 °C [26].

### 3.4. Characterization of DVS Cross-Linked Polymers

CHNS analyses were determined with a Thermo Scientific Flash 2000 elemental analyzer (Thermo Scientific, Madrid, Spain). Electron microscopy examination was performed on samples covered with gold using a sputter coater (SEMPREP2, Technoorg Linda LTD, Budapest, Hungary) and electron micrographs were taken with a S-510C scanning electron microscope at 3 kV (Hitachi High Technologies Europe GmbH, Krefeld, Germany). X-ray diffraction was carried out on a Pw1710/00 diffractometer (Philips, Madrid, Spain) using Cu Kα radiation. The operation voltage and current were 40 kV and 40 mA respectively. Data were collected from 3° to 80° with a 0.04° step and 0.4 s of integration. Data acquisition and processing were carried out with the Xpowder diffraction software [61]. IR spectra were recorded on a Spectrum Two (Perkin-Elmer, Madrid, Spain) and TGA analysis was carried out on dried polymers using a Shimadzu TGA-50H instrument (Shimadzu, Madrid, Spain) coupled to a Nicolet 550 IR-FT spectrometer (Thermo Scientific, Madrid, Spain). Samples were heated up to 950 °C in nitrogen at a heating rate of 20 °C.

### 3.5. Sorption Experiments

DVS cross-linked homo- or heteropolymer (100 mg) was added to different solutions (10 mL) of the phenolic compound or progesterone in water (for phenol or 4-nitrophenol, 20–5000 ppm) or 10% aqueous MeOH or DMSO (for β-naphthol, bisphenol A, 20–1000 ppm), in methanol for progesterone (5–50 ppm) or in absolute ethanol for curcumin (2–16.7 mM). The resulting suspensions were stirred in an orbital stirring table at 180 rpm for 3 h at room temperature. After this time, an aliquot of the starting solution and the treated solution were filtered in a syringe containing a regenerated cellulose filter (porous size 0.2 μm). The concentration of the filtered solutions was measured by UV/vis absorption in a Spectronic Unicam Heλios instrument (Thermo Fisher Scientific, Waltham, MA, USA) at 317 nm (phenol and 4-nitrophenol, 328 nm (β-naphthol, bisphenol A), 249 nm (progesterone) or at both 422 and 467 nm (curcumin).

## 4. Conclusions

Divinyl sulfone is an efficient cross-linking agent for the synthesis of aqueous insoluble cyclodextrin-based polymers (CDPs) and other insoluble polymers from polysaccharides, providing that the reaction is carried out at an alkaline pH. The degree of cross-linking exerts a clear influence on the surface of the material, whereas it plays a minor role on the formation of the inclusion complexes. In general, inclusion experiments on the CDPs are better described by the Freundlich isotherm although an important number of them can also be fitted to the Langmuir isotherm with R^2^ ≥ 0.9, suggesting a sorption onto a monolayer of homogeneous sites. The results demonstrate the versatility of CDPs and highlight the important role of the CDs, supporting a sorption process based on the formation of inclusion complexes that may explain the poor performance of the cross-linked starch used as reference.

## Supplementary Materials

Supplementary materials can be accessed at: http://www.mdpi.com/1420-3049/20/03/3565/s1.
