# Supplementary material for: Divinyl Sulfone Cross-Linked Cyclodextrin-Based Polymeric Materials: Synthesis and Applications as Sorbents and Encapsulating Agents
Source: Molecules. 2015 Feb 19;20(3):3565–81. doi: 10.3390/molecules20033565 (PMC6272569; doi:10.3390/molecules20033565)
Supplement: Supplementary file 1 [file molecules-20-03565-s001.pdf]

Supplementary Materials

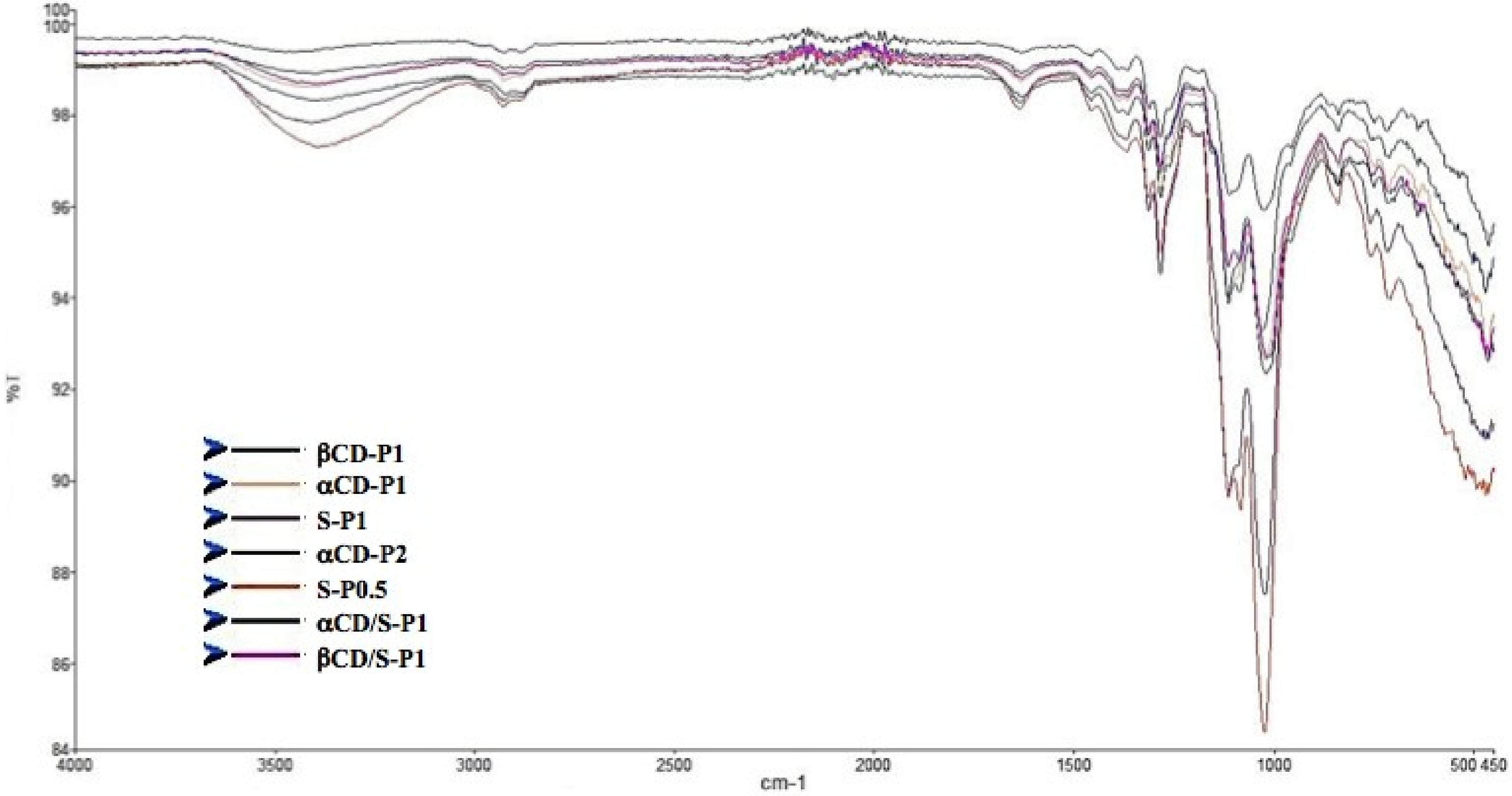

Figure S1. ATR-IR spectra for homo- and hetero-CDPs.

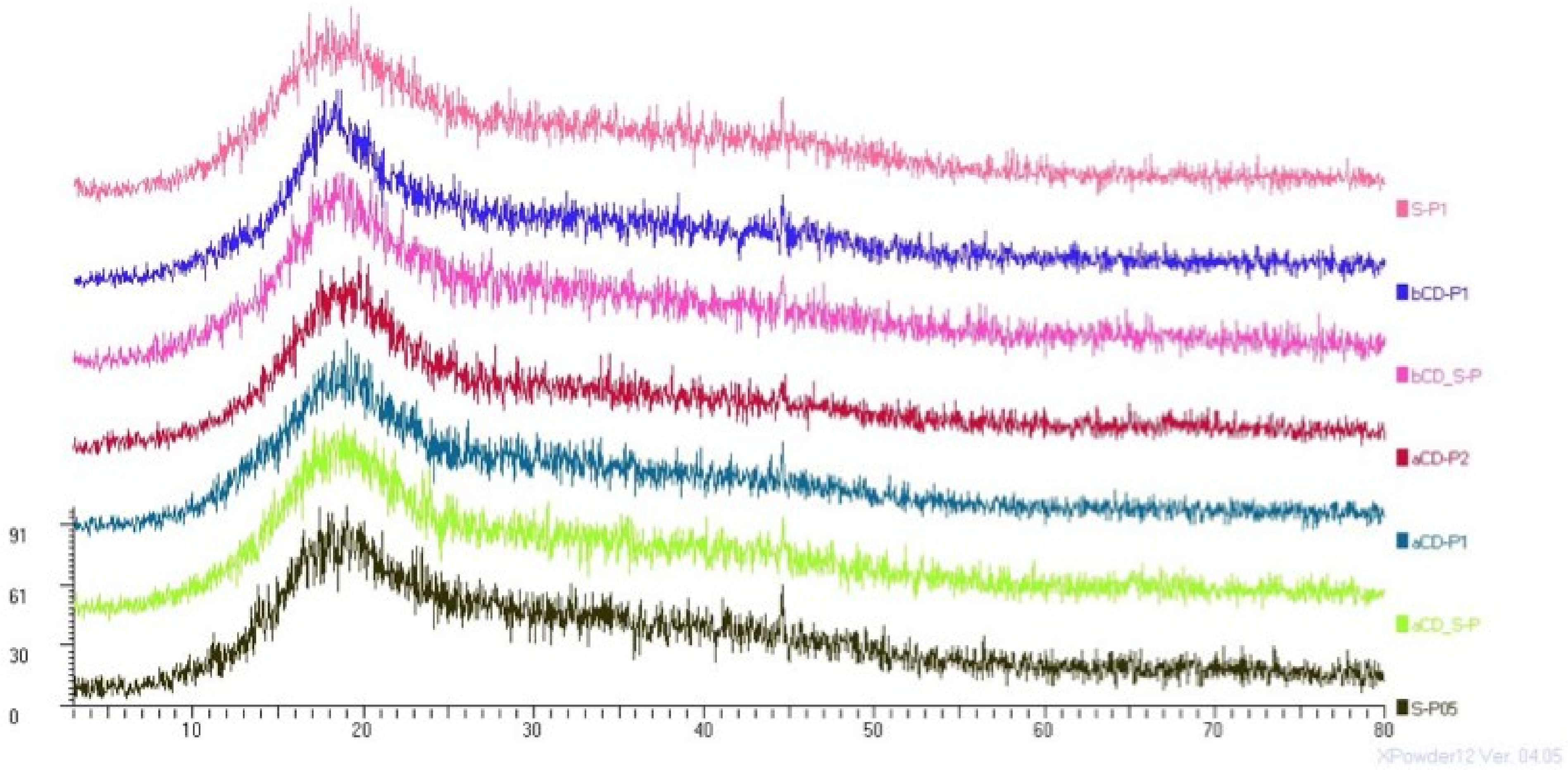

Figure S2. X-ray diffraction pattern for the polymers resulting from homo- and hetero DVS cross-linked CDPs.

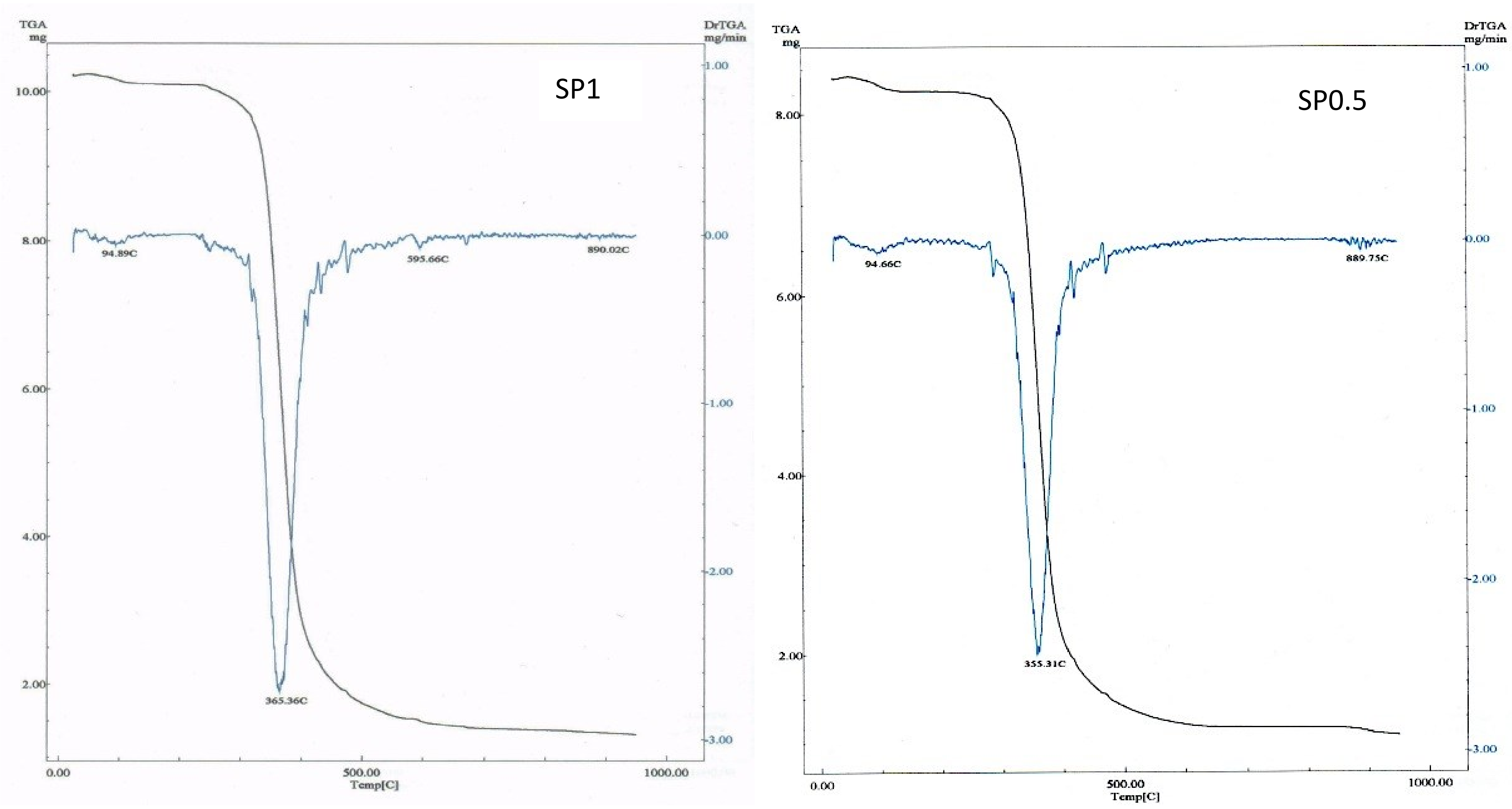

Figure S3. *Cont.*

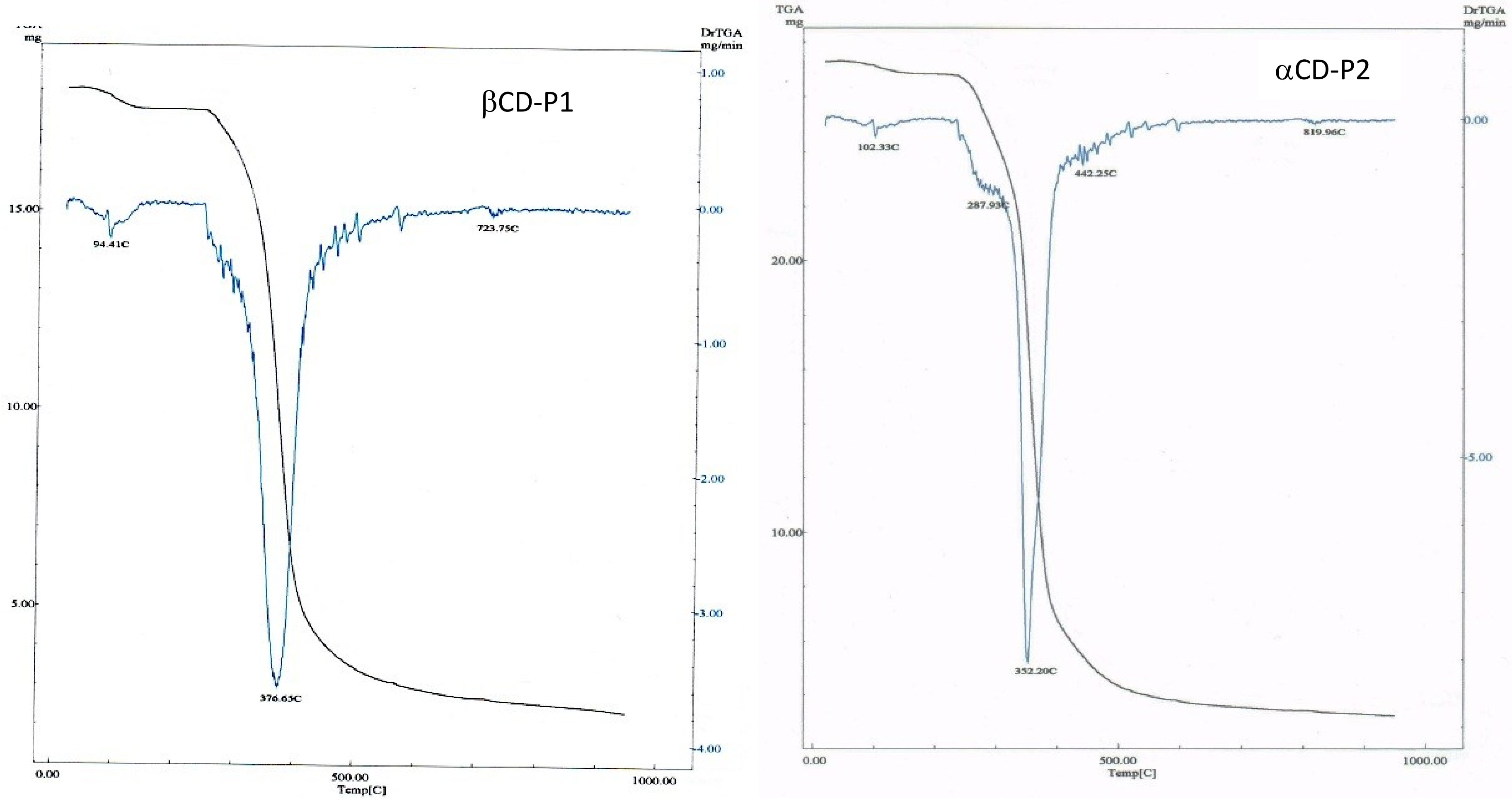

Figure S3. *Cont.*

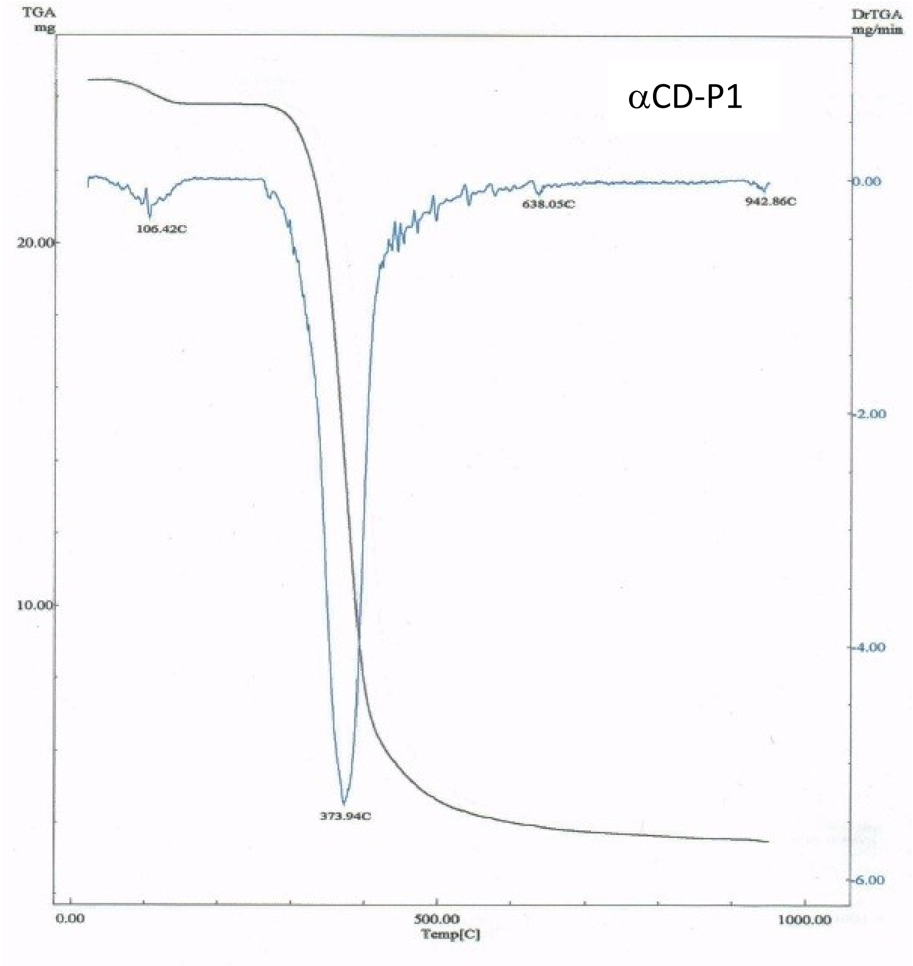

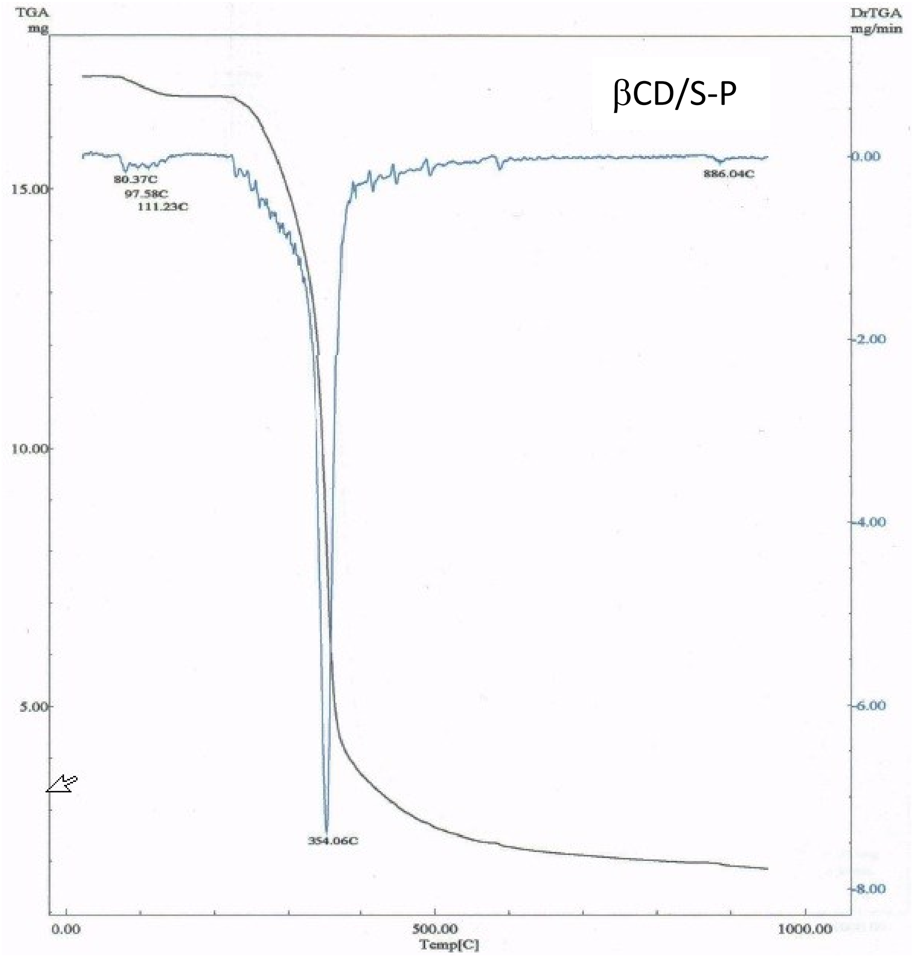

Figure S3. *Cont.*

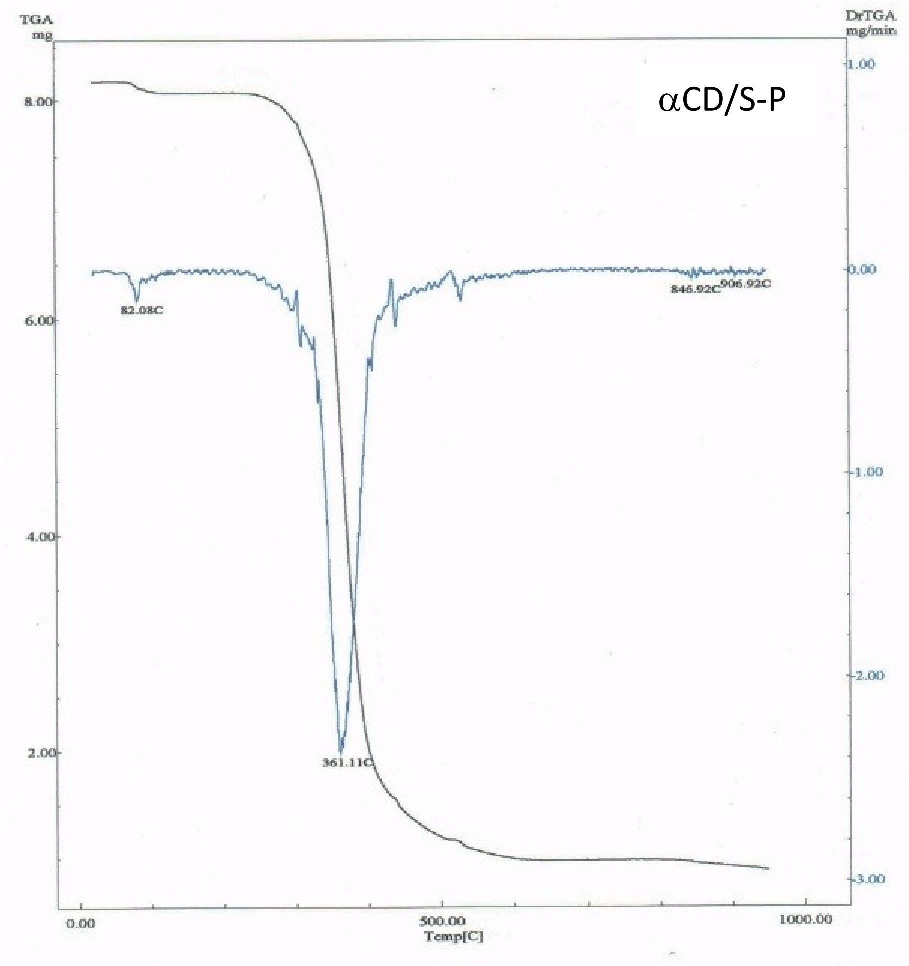

Figure S3. TGA for homo- and hetero-polymers.

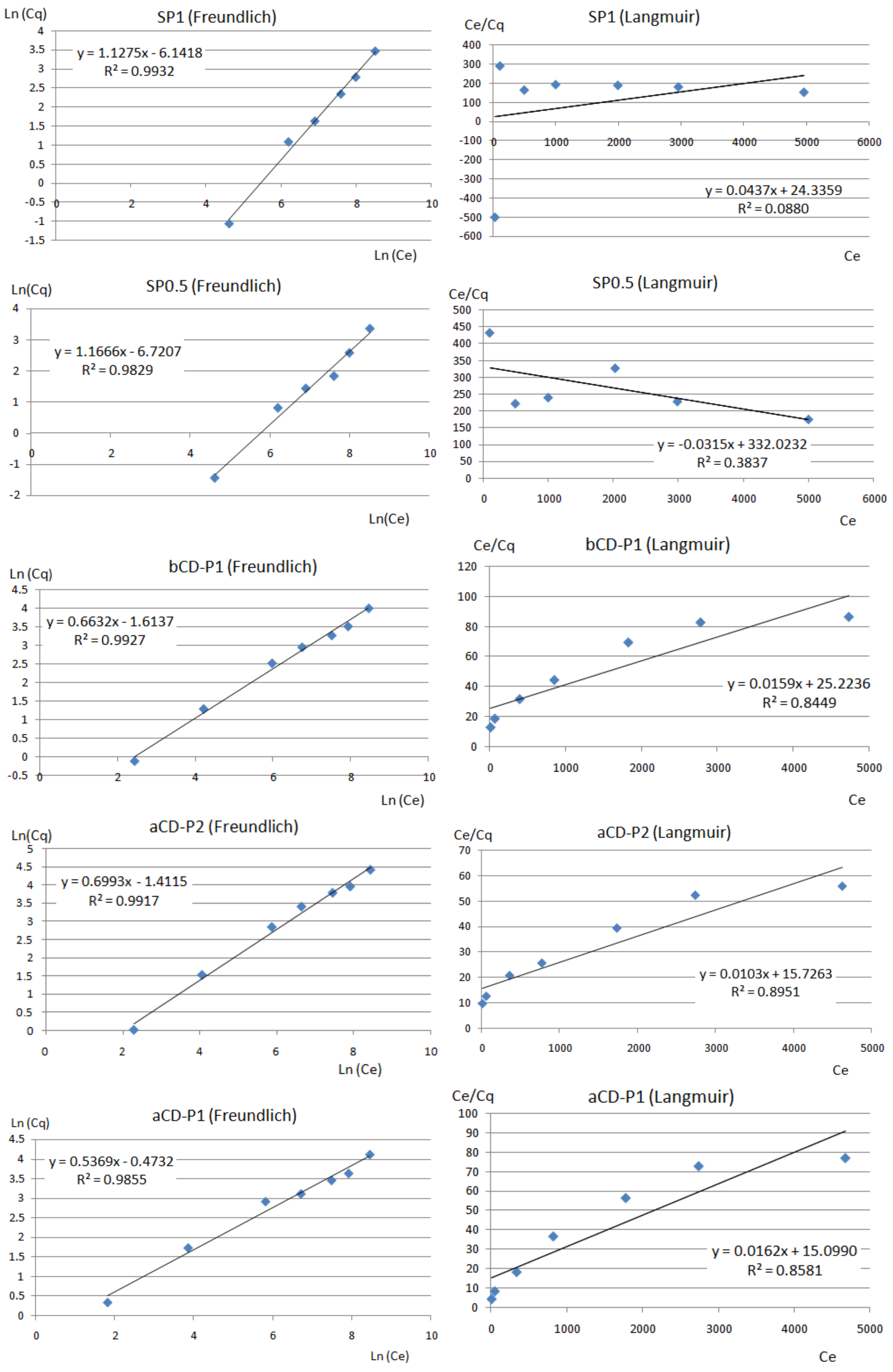

Figure S4. *Cont.*

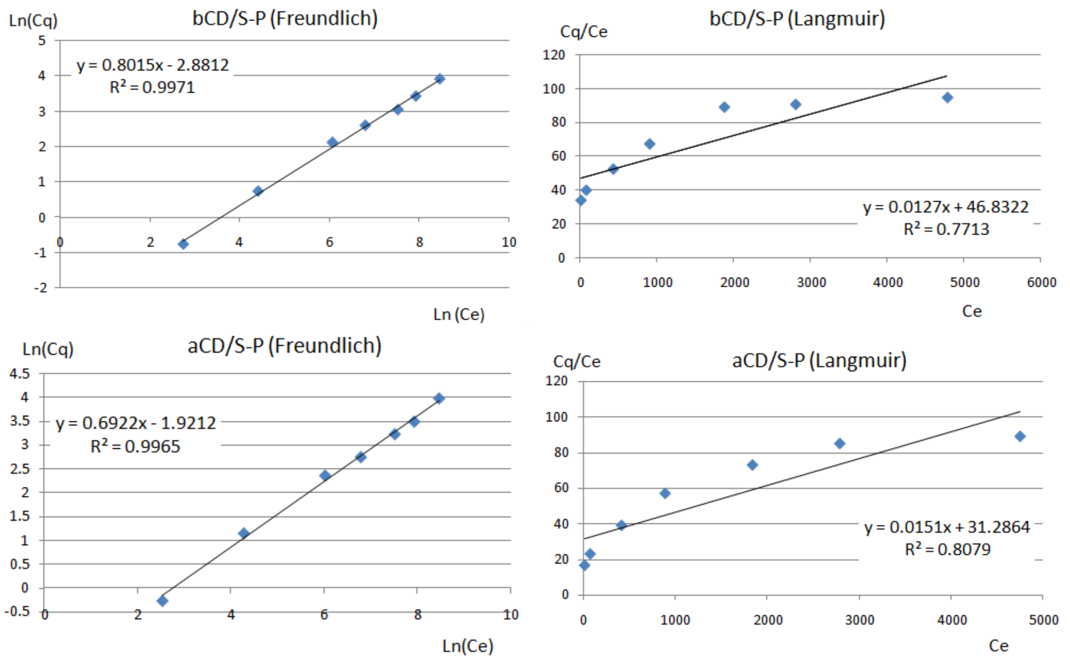

Figure S4. Sorption of phenol (20–5000 ppm in water) on the polymers (100 mg). Fitting of the experimental data to the linearized forms of the isotherms of Freundlich and Langmuir.

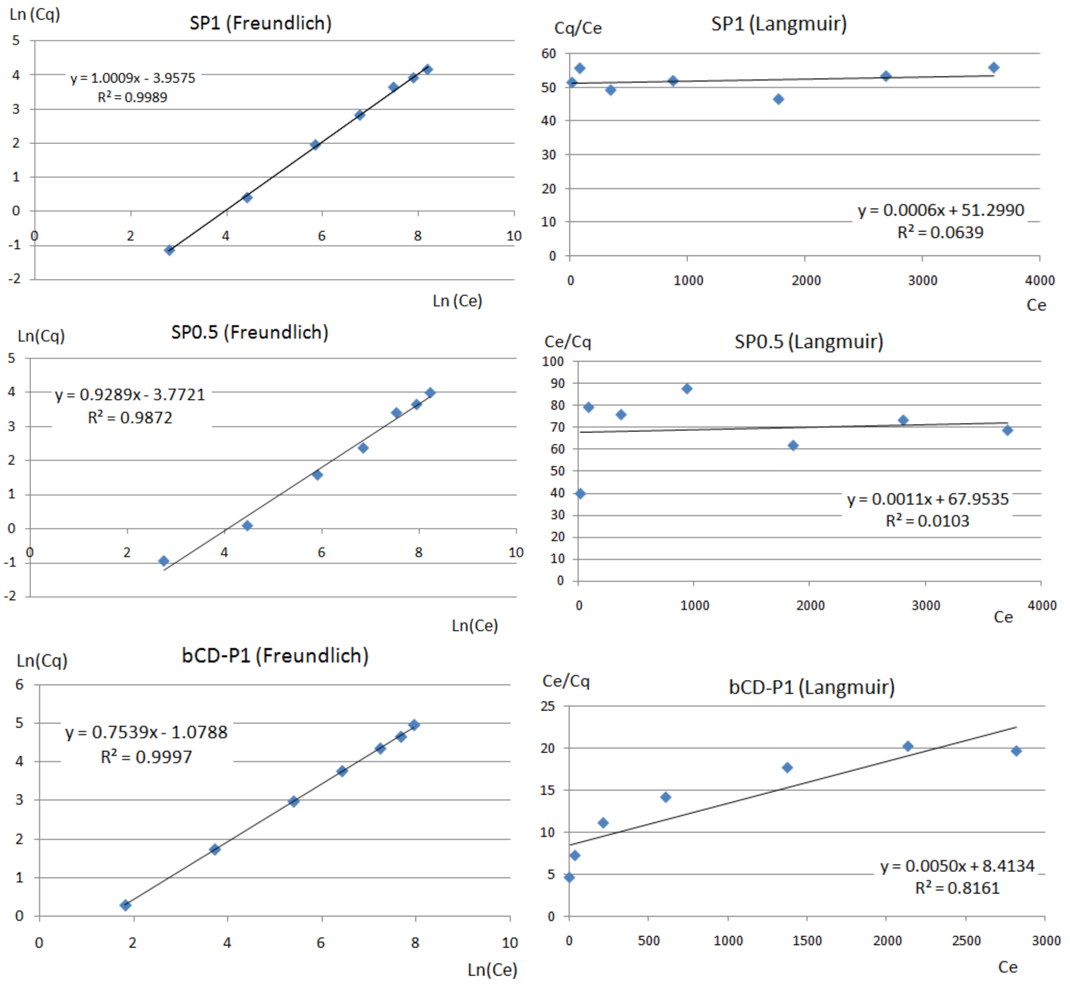

Figure S5. *Cont.*

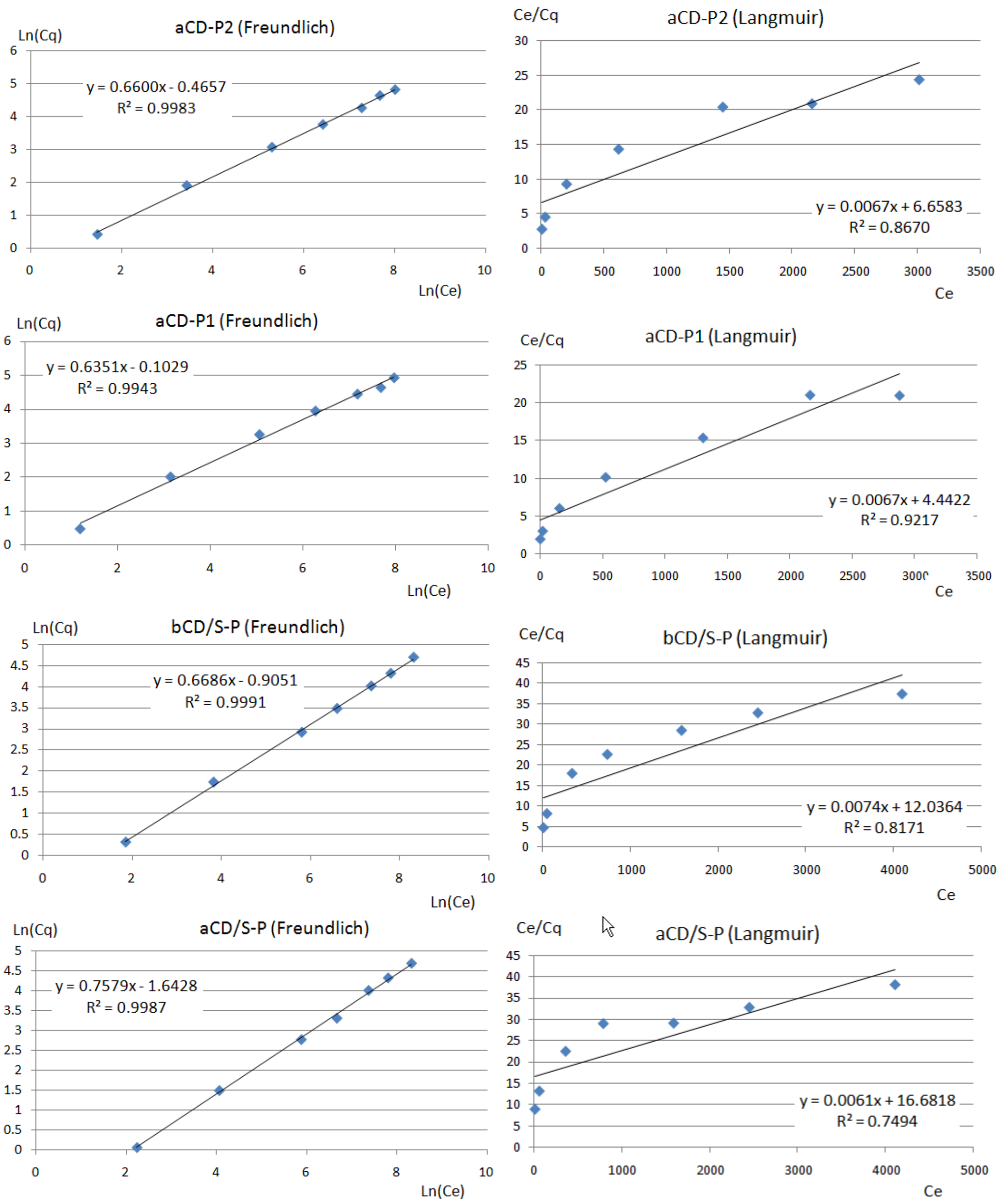

Figure S5. Sorption of p-nitro-phenol (20–5000 ppm in water) on the polymers (100 mg). Fitting of the experimental data to the linearized forms of the isotherms of Freundlich and Langmuir.

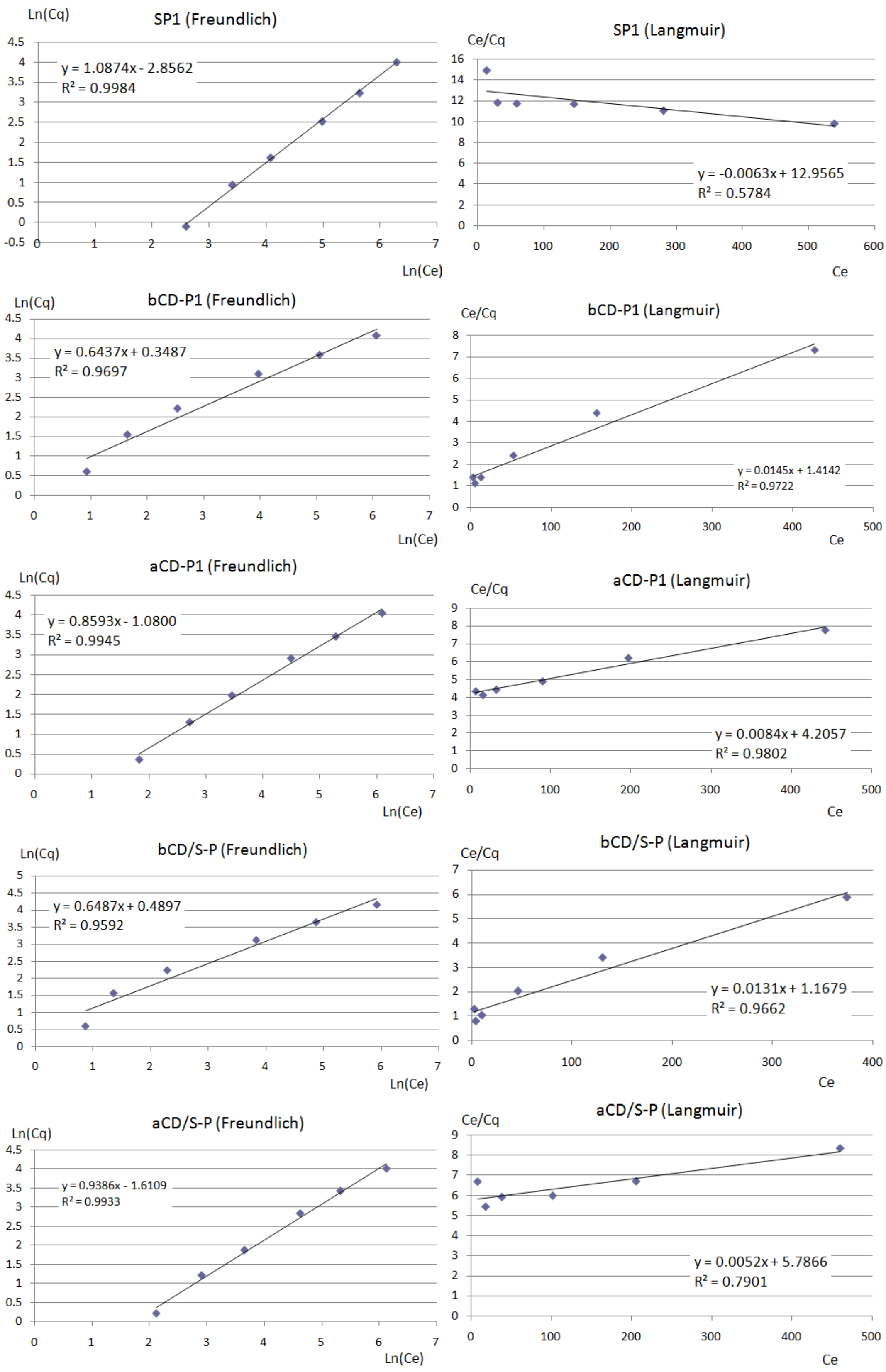

Figure S6. Sorption of bisphenol (20–1000 ppm in methanol: water 1:9) on the polymers (100 mg). Fitting of the experimental data to the linearized forms of the isotherms of Freundlich and Langmuir.

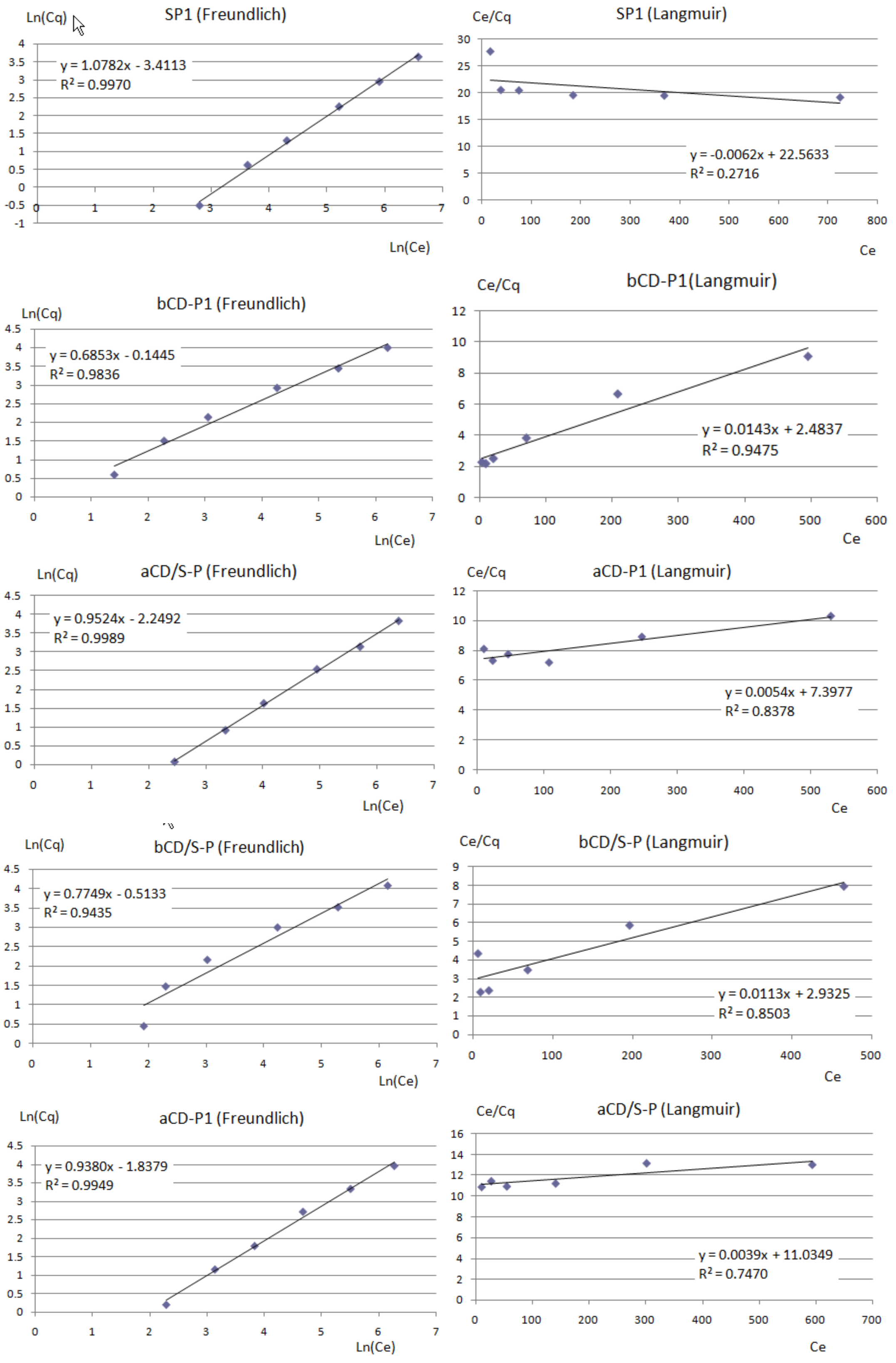

Figure S7. Sorption of bisphenol (20–1000 ppm in DMSO: water 1:9) on the polymers (100 mg). Fitting of the experimental data to the linearized forms of the isotherms of Freundlich and Langmuir.

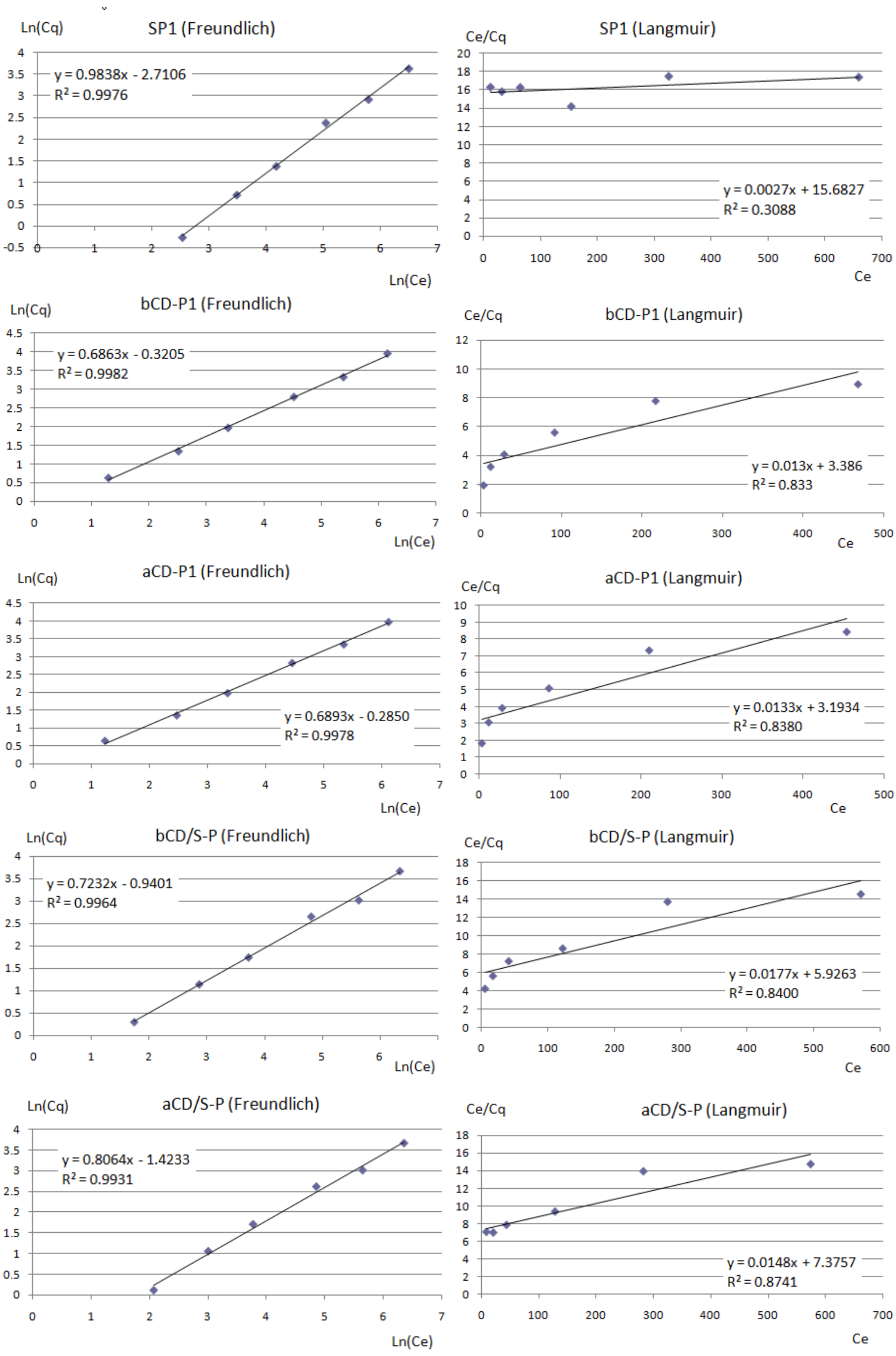

Figure S8. Sorption of β-naphtol (20–1000 ppm in methanol:water 1:9) on the polymers (100 mg). Fitting of the experimental data to the linearized forms of the isotherms of Freundlich and Langmuir.

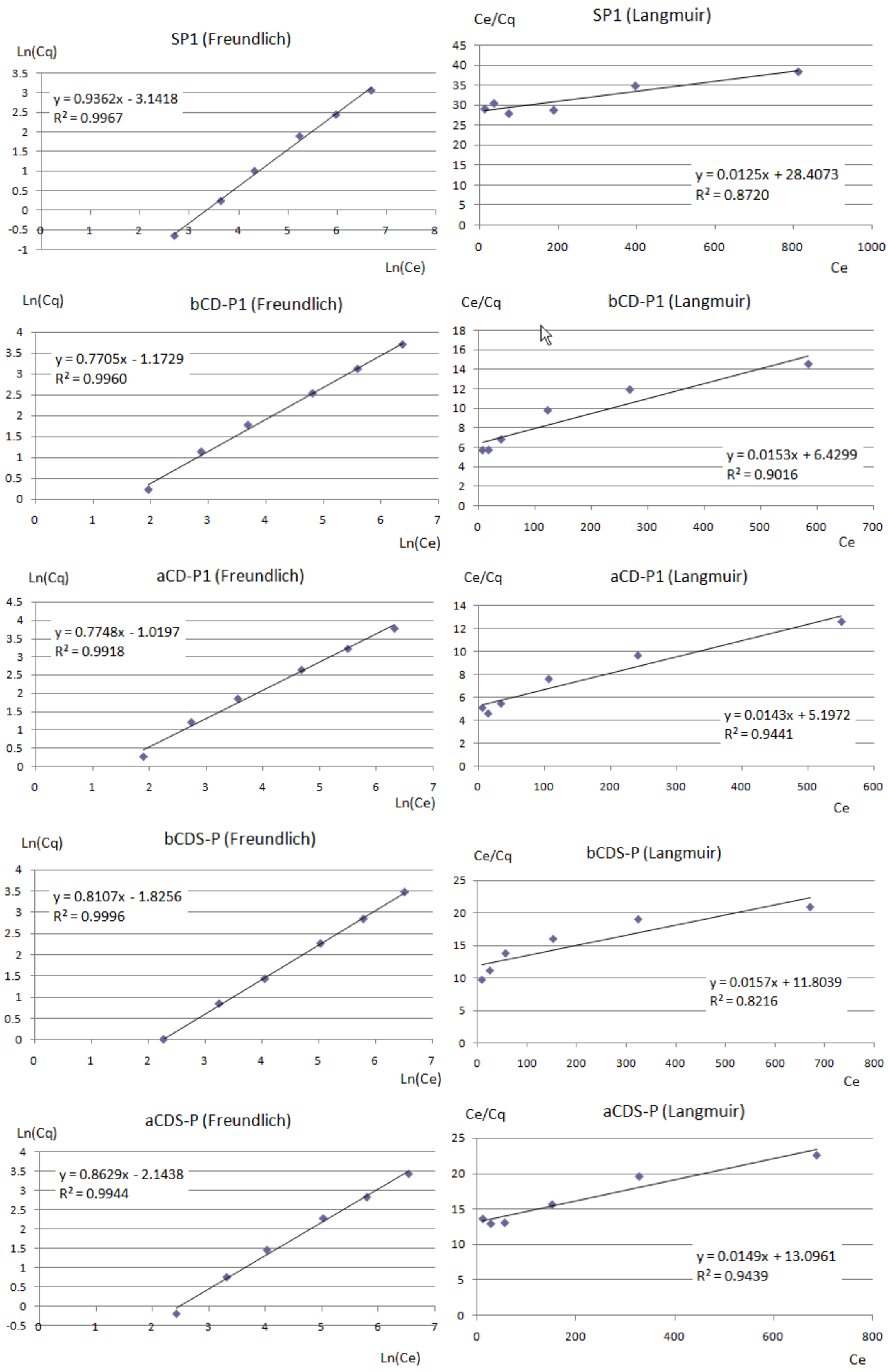

Figure S9. Sorption of β-naphtol (20–1000 ppm in DMSO:water 1:9) on the polymers (100 mg). Fitting of the experimental data to the linearized forms of the isotherms of Freundlich and Langmuir.

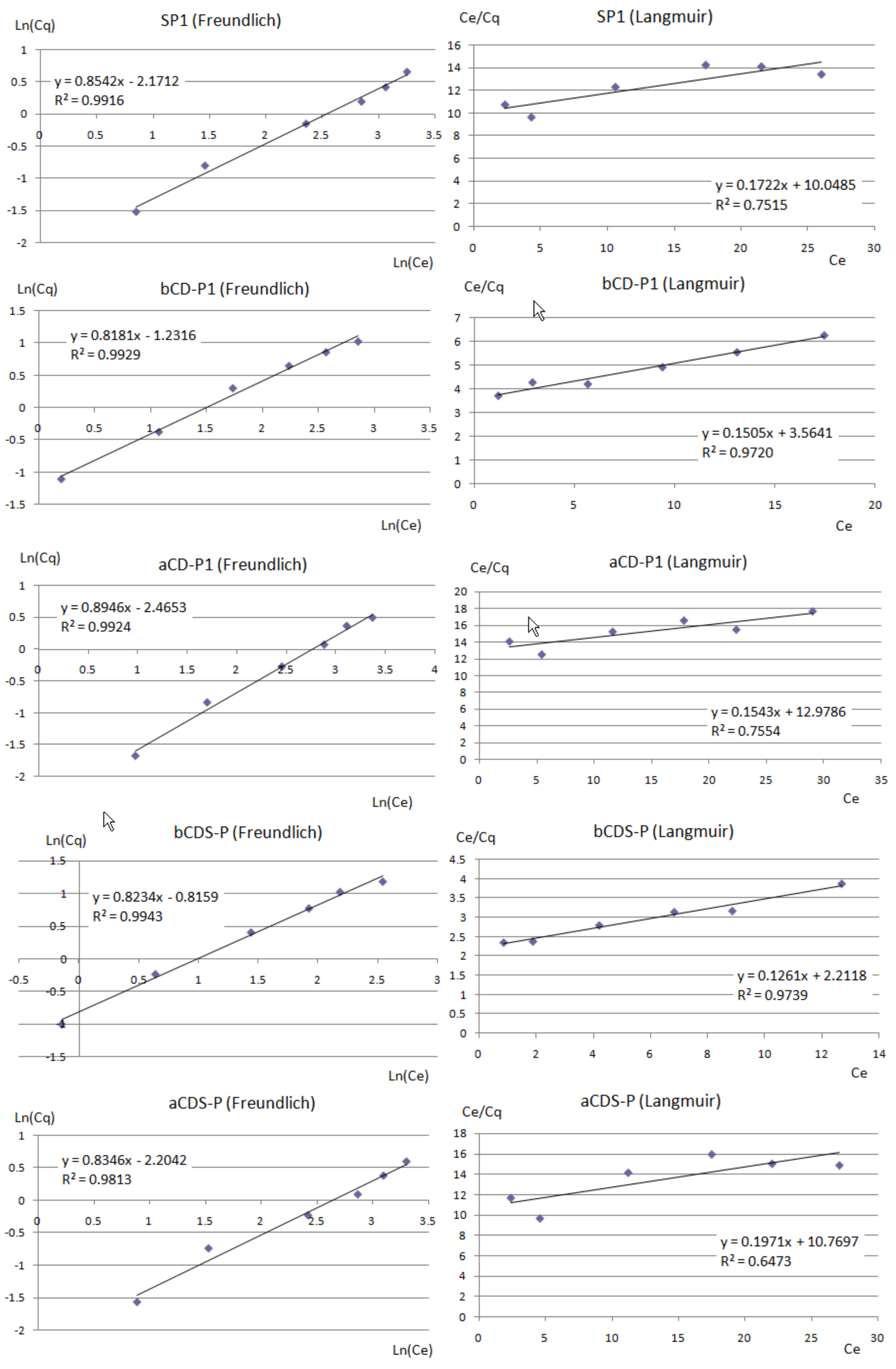

Figure S10. Sorption of progesterone (5–50 ppm in methanol:water 1:9) on the polymers (100 mg). Fitting of the experimental data to the linearized forms of the isotherms of Freundlich and Langmuir.

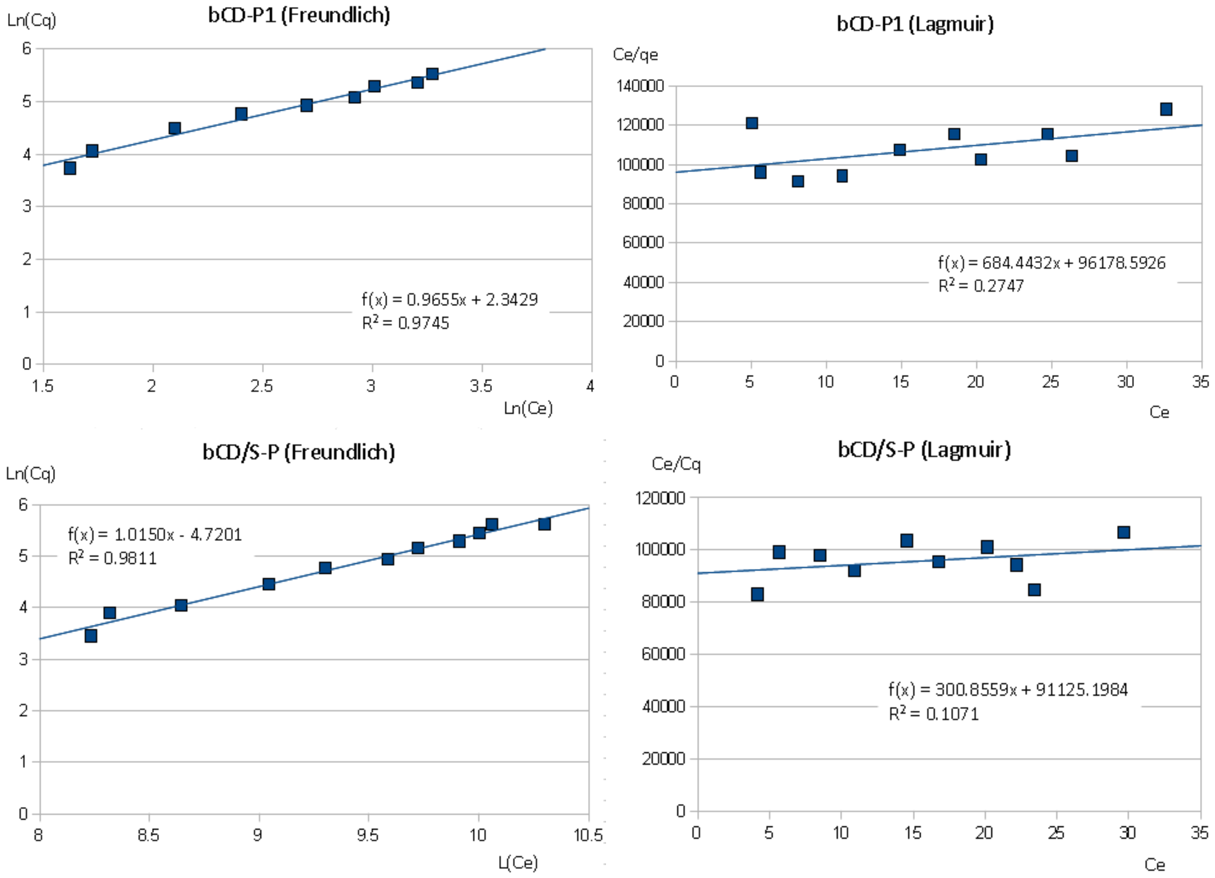

Figure S11. Sorption of curcumin (2–16.7 mM in ethanol) on the polymers (125 mg). Fitting of the experimental data to the linearized forms of the isotherms of Freundlich and Langmuir.
